# The Clinical Significance of Femoral and Tibial Anatomy for Anterior Cruciate Ligament Injury and Reconstruction

**DOI:** 10.2174/0115734056361050250605052447

**Published:** 2025-06-19

**Authors:** Junqing Liang, Fong Fong Liew

**Affiliations:** 1 Department of Sports Medicine, Baise People's Hospital, Guangxi, China; 2 Faculty of Medicine, MAHSA University, Jenjarom, Selangor, Malaysia; 3 Department of Preclinical Sciences, Faculty of Dentistry, MAHSA University, Jenjarom, Selangor, Malaysia

**Keywords:** Anterior cruciate ligament, ACL, Intercondylar notch, Tibial tuberosities, Injury prevention, Injury management, Patient recovery

## Abstract

The anterior cruciate ligament (ACL) is a crucial stabilizer of the knee joint, and its injury risk and surgical outcomes are closely linked to femoral and tibial anatomy. This review focuses on current evidence on how skeletal parameters, such as femoral intercondylar notch morphology, tibial slope, and insertion site variations—influence ACL biomechanics. A narrowed or concave femoral notch raises the risk of impingement, while a higher posterior tibial slope makes anterior tibial translation worse, which increases ACL strain. Gender disparities exist, with females exhibiting smaller notch dimensions, and hormonal fluctuations may contribute to ligament laxity. Anatomical changes that come with getting older make clinical management even harder. Adolescent patients have problems with epiphyseal growth, and older patients have to deal with degenerative notch narrowing and lower bone density. Preoperative imaging (MRI, CT, and 3D reconstruction) enables precise assessment of anatomical variations, guiding individualized surgical strategies. Optimal femoral and tibial tunnel placement during reconstruction is vital to replicate native ACL biomechanics and avoid graft failure. Emerging technologies, including AI-driven segmentation and deep learning models, enhance risk prediction and intraoperative precision. Furthermore, synergistic factors, such as meniscal integrity and posterior oblique ligament anatomy, need to be integrated into comprehensive evaluations. Future directions emphasize personalized approaches, combining advanced imaging, neuromuscular training, and artificial intelligence to optimize prevention, diagnosis, and rehabilitation. Addressing age-specific challenges, such as growth plate preservation in pediatric cases and osteoarthritis management in the elderly, will improve long-term outcomes. Ultimately, a nuanced understanding of skeletal anatomy and technological integration holds promise for reducing ACL reinjury rates and enhancing patient recovery.

## 1 INTRODUCTION

The Anterior Cruciate Ligament (ACL) is a primary stabilizer of the knee joint, preventing anterior tibial translation and internal rotation, and is essential for normal knee biomechanics [1, 2]. ACL injuries, common in sports, often result in knee instability and reduced activity, frequently requiring surgical intervention. Fig. (**1**) illustrates arthroscopic views of a normal and ruptured ACL, while Fig. (**2**) provides an MRI sagittal view of a healthy ACL, offering critical diagnostic insights into its integrity and positioning.

ACL ruptures are classified into three categories: (1) subsynovial tears/strains and proximal avulsion tears, (2) single-bundle tears (anteromedial/posterolateral), and (3) complete tears. Studies show that femoral and tibial anatomical structures significantly influence ACL position, length, and cross-sectional area, affecting its stability and injury risk [3-5]. These anatomical parameters are therefore essential considerations in preoperative surgical planning and significantly impact the success of ACL reconstruction outcomes [6-10]. Consequently, a thorough understanding of the intricate relationship between ACL pathology and the surrounding bony structures of the femur and tibia is paramount for advancing our knowledge of ACL injury mechanisms, refining surgical techniques, and ultimately improving patient outcomes.

Beyond the physical challenges, ACL injuries also impose a substantial financial burden on patients, particularly those with high-deductible health plans, even amidst declining insurance reimbursements [11, 12]. Furthermore, the long-term consequences of untreated ACL injuries extend beyond pain and financial strain, significantly increasing the risk of developing osteoarthritis, which can lead to chronic pain, joint dysfunction, diminished quality of life, and escalating long-term healthcare costs [13]. These consequences underscore the need for in-depth research to prevent complications and improve patient outcomes.

The significance of this study lies in its potential to bridge gaps in current ACL injury management by elucidating key anatomical risk factors and their role in injury susceptibility. Understanding the relationship between ACL pathology and anatomical structures such as the femoral intercondylar notch and tibial tuberosities is crucial for identifying individuals at higher risk for ACL injuries. This knowledge can contribute to improved screening, prevention, and rehabilitation strategies tailored to specific anatomical variations. Additionally, by synthesizing current research, this study aims to refine surgical reconstruction techniques, enhancing better graft placement, biomechanical stability, and overall success in ACL repair.

Moreover, as ACL injuries frequently impact young athletes and active individuals, optimizing prevention strategies is essential in mitigating long-term morbidity. By focusing on anatomical and biomechanical contributors to ACL injuries, this study can enhance clinical decision-making, allowing for more personalized treatment approaches that reduce reinjury rates and improve long-term knee function. In addition, this study seeks to improve personalized approaches to ACL injury prevention, diagnosis, and surgical management, thereby improving patient outcomes and reducing healthcare burdens associated with ACL pathology.

## 2 SKELETAL ANATOMY AND THE BIOMECHA-NICAL MECHANISMS OF ACL INJURY

### 2.1 The Influence of the Femoral Intercondylar Notch on ACL Injury Risk

The femoral intercondylar notch, the bony indentation where the ACL originates on the thigh bone (femur), plays a crucial role in the ACL's function and susceptibility to injury. Studies have shown that abnormalities in notch shape and size, particularly a smaller volume and narrowing, can increase the risk of ACL fibers impinging on the notch walls, leading to tears [9, 10]. Females tend to have narrower intercondylar notches than males, a factor that may contribute to the higher incidence of ACL injuries in female athletes. Combinations of knee joint geometry measurements provide more insight into the risk of noncontact ACL injury than individual parameters. The aspects of knee geometry most strongly associated with injury risk differ between males and females. For example, a female with both a decreased femoral notch width and an increased posterior-inferior–directed lateral compartment tibial articular cartilage slope, or a male with both a decreased ACL volume and a reduced lateral compartment posterior meniscus angle, is at the highest risk for ACL injury [14]. This increased risk is thought to stem from the reduced space within the intercondylar notch, which may predispose the ACL to impingement against the femoral condyles during dynamic knee movements such as pivoting or landing from a jump. Furthermore, a lower notch shape index has been linked to altered biomechanical loading on the ACL, increasing its susceptibility to rupture under stress [15, 16]. Future research should further explore the interplay between notch morphology, ACL biomechanics, and injury mechanisms to optimize preventive strategies and improve clinical outcomes.

The morphology of the femoral intercondylar notch is a critical factor in ACL injuries. Dias *et al.* proposed using specific curvature descriptors of the notch to assess ACL injury risk, suggesting that greater narrowing or concavity is associated with a higher risk [17]. Similarly, Sangeeta *et al.* identified a negative correlation between the notch width-to-height ratio and the ACL's anatomical length and cross-sectional area, indicating that narrower notches may predispose individuals to impingement injuries [18]. Emerging technologies, particularly deep learning-based automatic segmentation models, show great promise in refining the measurement of notch volume and shape. These models provide precise, reliable data for both ACL tunnel placement during reconstructive surgery and injury risk prediction [19-21]. By leveraging these advancements, clinicians can enhance the accuracy of risk assessments and surgical planning.

During surgery, the ACL's proximal attachment is visualized on the medial side of the lateral femoral condyle. This region occupies approximately two-thirds of the condyle's upper medial portion, as shown in Fig. (**3**). The overall femoral attachment site of the ACL can be precisely located using four readily identifiable arthroscopic landmarks: the lateral intercondylar ridge, the bifurcate ridge, the posterior cartilage border, and the distal cartilage border [22]. Measuring the intercondylar notch’s three-dimensional geometric parameters, such as volume, height-to-width ratio, and curvature-provides valuable insights into the ACL’s anatomical trajectory and potential for injury.

Importantly, most previous studies relied on two-dimensional or semi-automatic three-dimensional measurement techniques, which are prone to subjectivity and errors. Fortunately, advancements in imaging technology have paved the way for fully automated segmentation and measurement methods powered by deep learning algorithms. These methods offer greater objectivity and accuracy, representing a significant step forward in ACL injury risk assessment.

### 2.2 Tibial Insertion Site Variations and their Impact on ACL Function

The tibial insertion site of the ACL is a critical factor in ACL reconstruction. Accurate graft placement within the tibial tunnel is essential to restoring proper knee mechanics and minimizing the risk of graft failure. Variations in the tibial insertion site can lead to imbalances in graft tension, affecting knee stability and function [23]. Anatomical placement of the tibial insertion is crucial for replicating the native ACL's biomechanical function, ensuring optimal knee stability, and reducing the risk of re-injury.

Variations in the ACL’s tibial insertion site, as well as surrounding cartilage and bone structures, can significantly influence ligament positioning and biomechanics [24]. Yonetani *et al.* [25] and Gulan *et al.* [26] conducted detailed anatomical studies examining the ACL’s tibial insertion site in adults and its relationship to other tibial structures. Their findings revealed that the insertion site typically lies between the medial and lateral tibial plateaus, with a slight lateral bias for the anteromedial bundle and a slight medial bias for the posterolateral bundle, as depicted in Fig. (**4**). Additionally, the tibial intercondylar eminence slopes downward and laterally below the insertion, creating a “support slope” for the ACL [25-27]. In children and adolescents, incomplete development of bone growth plates (epiphyses) can result in positional variations of the ACL’s tibial insertion site [28]. These anatomical differences have important implications for both accurately locating the ACL insertion and selecting appropriate tibial tunnel drilling techniques during reconstructive surgery.

The tibial slope, particularly the posterior tibial slope, plays a critical role in ACL biomechanics. In a study involving 50 patients with a third ACL injury, 24 reinjured the knee that had undergone revision ACL reconstruction, while 26 injured the contralateral knee. The posterior tibial slope was significantly greater in the third-injury group compared to those without a third injury (medial: 7.5° *vs*. 6.3°; lateral: 13.6° *vs*. 11.9°). This suggests that an increased posterior tibial slope, especially on the lateral tibial plateau, is a key risk factor for recurrent ACL injuries, including both graft ruptures and contralateral ACL tears [29].

Injuries near the ACL’s tibial insertion site can also compromise its function. Fractures of the tibial intercondylar eminence and tibial tuberosity, for example, increase the risk of ACL tears, particularly in younger individuals [30]. Furthermore, during ACL reconstruction, the placement, approach, and angle used for drilling the tibial tunnel significantly impact the preservation and restoration of function at the ACL insertion site [6, 8]. Therefore, a thorough preoperative evaluation of bone integrity (osseous status) in the tibial insertion area using imaging techniques is crucial for determining the optimal tunnel placement angle and drilling technique.

### 2.3 Skeletal Parameters and ACL Structural Characteristics (Length, Area and Signal Intensity)

Structural parameters of the ACL, such as length and cross-sectional area, directly affect its load-bearing capacity and biomechanical function. Numerous studies have explored the correlation between osseous landmarks and ACL structural parameters, providing insights into the pathophysiological mechanisms of ACL function and injury risk [31, 32].

The length of the ACL is a critical factor in its ability to withstand tensile stress. Blumensaat’s line, a radiographic landmark drawn along the roof of the femoral intercondylar notch, has been shown to correlate positively with ACL length [33]. This correlation allows for the estimation of ACL length based on Blumensaat’s line. However, studies by Tran *et al*. [34] and others suggest that this correlation may not be reliable in children and adolescents due to ongoing skeletal development. Additionally, ACL length exhibits gender differences, with females typically having shorter ACLs than males [35]. These findings highlight the potential of Blumensaat’s line for preoperative ACL length assessment in adults while emphasizing the need for additional anatomical landmarks to improve accuracy in younger patients.

The cross-sectional area of the ACL is a key parameter for evaluating its load-bearing capacity. Studies by Barnett *et al*. [36] and Wang *et al*. [37] identified a positive correlation between the ACL area and the size and shape of the lateral wall of the femoral intercondylar notch. This suggests that femoral anatomy influences the ACL’s blood supply, ultimately affecting its size and ability to bear loads. Furthermore, studies indicate that adult males have significantly larger ACL areas compared to females, and children have smaller ACL areas overall [35, 36]. Research by Iriuchishima *et al.* also revealed a positive correlation between the ACL area at its femoral attachment site and an individual’s height and weight. Collectively, these findings provide a theoretical foundation for assessing ACL load-bearing capacity based on patient characteristics [38]. Factors affecting ACL signal intensity in medical imaging have also garnered research interest. Increased signal intensity has been suggested as a potential indicator of insufficient blood supply to the ACL, with femoral anatomy playing a significant role in ACL vascularization. Patients with femoral abnormalities, such as a narrowed intercondylar notch, may exhibit abnormally high ACL signal intensity, potentially indicating early signs of ACL degeneration or pathology. While preliminary research suggests a possible link between ACL cross-sectional area, signal intensity, and injury risk, further investigation is needed to determine the relative importance of each factor in quantitatively assessing ACL health and injury potential [36].

The morphology of the tibial plateau, including the width and surface area of the medial and lateral plateaus, also influences ACL function by affecting load distribution across the knee joint. In particular, the posterior tibial slope plays a crucial role in ACL biomechanics and injury susceptibility. An increased posterior tibial slope, especially on the lateral side, significantly impacts knee biomechanics by enhancing anterior tibial translation during dynamic movements such as running, jumping, and cutting. This forward motion places additional stress on the ACL, increasing its strain and elevating the risk of injury or graft failure, particularly after ACL reconstruction surgery [39, 40]. In terms of correlation with ACL structural parameters, a steeper tibial slope may also influence ACL length and cross-sectional area, as these factors determine the ligament’s ability to bear load and resist mechanical stress. Studies suggest that this biomechanical relationship is an important consideration when evaluating injury risk and predicting long-term outcomes following ACL surgery [40, 41].

### 2.4 The Association between ACL Graft Tunnel Positioning and Skeletal Anatomy

In post-ACL reconstruction surgery, accurately selecting the positions of the femoral and tibial tunnels is crucial for replicating the ACL’s anatomical path, preventing graft impingement on bone structures, and optimizing motion recovery. Properly placed femoral tunnels are located in the low posterior position of the lateral femoral condyle, guided by the lateral intercondylar ridge and the bifurcate ridge. The tibial tunnel is centered based on the posterior edge of the medial tibial spine and the anterior horn of the lateral meniscus, aiming to restore 50% to 80% of the tibial footprint area. Additionally, measuring the intercondylar notch width is critical for predicting graft size and preventing impingement, ultimately helping to restore the knee joint’s original biomechanics [42].

The shape and size of the femoral intercondylar notch directly influence the drilling approach and optimal femoral tunnel placement [7, 43]. Studies using dynamic models have shown that improperly positioned femoral tunnels increase the risk of graft impingement against the notch walls, leading to excessive mechanical stress and a higher likelihood of graft failure or ACL re-tear [44]. In order to address this, Li *et al.* proposed using the posterior lateral trochlear edge as a reference point for femoral tunnel placement to avoid impingement zones [45]. Research also suggests that traditional lateral approaches for drilling femoral tunnels often result in tunnels longer than expected, potentially due to abnormal intercondylar notch anatomy [46]. Advanced imaging techniques, such as dual C-arm live imaging and 3D CT reconstruction, can be used postoperatively to quantify the extent of graft exposure within the intercondylar notch [47, 48]. These precise measurement methods hold promise for refining surgical techniques further.

The approach and positioning of the tibial tunnel significantly impact the ACL graft's functional recovery. To achieve optimal outcomes, surgeons aim to replicate the ACL's original anatomical position within the tibia while avoiding nearby bone structures [6]. A thorough evaluation of tibial structures, including the intercondylar eminence and tibial tubercle morphology, along with 3D imaging analysis, is essential for determining the tunnel angle and any necessary deviations from a perfectly straight path [8]. Research has shown that postoperative complications such as synovial sheath formation and bone cysts can indicate tunnel misplacement [49]. In a study of 60 ACL reconstructions with an average follow-up of 34 months, no significant difference was observed in tibial tunnel positioning between two femoral tunnel drilling techniques: technique A, where the femoral tunnel was drilled through a far medial portal while viewing from a high anterolateral portal, and technique B, where the tunnel was drilled through a far medial portal while viewing through a high anteromedial portal. However, patients with well-placed femoral tunnels had significantly higher Lysholm (62.2 ± 16.2 *vs*. 48.5 ± 17.2, p = 0.002) and The International Knee Documentation Committee (IKDC) scores (62.5 ± 14.3 *vs*. 52.7 ± 15.1, p = 0.012). Additionally, those who underwent surgery within 3 months of injury had better hop test results (4.4 ± 0.9 *vs*. 3.9 ± 1, p = 0.034) and IKDC scores (62.5 ± 15.8 *vs*. 33.2 ± 13.8, p = 0.026) compared to those who delayed surgery. The study concludes that well-placed femoral tunnels and timely surgery within 3 months of injury lead to better clinical outcomes [50, 51]. Therefore, meticulous preoperative imaging assessment is essential to identify and avoid anatomical obstacles that could compromise graft function and long-term knee stability.

## 3 THE CLINICAL SIGNIFICANCE OF ANATOMICAL VARIATIONS

### 3.1 Gender Differences and Hormonal Influences

Studies have reported how anatomical features of the ACL change during bone growth and maturation, potentially influencing injury risk. Research reveals significant age-related variations, with distinct differences between males and females. During puberty, males exhibit increased ACL length and thickness, whereas females have smaller ACLs with a slower rate of cross-sectional growth. This disparity in ACL morphology may contribute to the higher incidence of ACL injuries observed in females. Additionally, changes in ACL anatomy linked to skeletal maturity differ between genders, potentially explaining age- and sex-related variations in ACL injury prevalence [52, 53]. However, this retrospective cross-sectional study has limitations, including patient selection bias and the inability to capture longitudinal changes. Age may not accurately reflect skeletal maturity, and other factors such as ethnicity, activity level, and genetics may also influence ACL morphology.

Research suggests that hormonal fluctuations, particularly over the menstrual cycle, may affect ligamentous laxity in females. Elevated estrogen and relaxin levels have been linked to increased ligament laxity, potentially raising the risk of ACL injuries in female athletes. The biomechanical response of the ACL may fluctuate throughout the cycle, influencing joint stability and susceptibility to injury, especially during high-demand activities [54, 55].

However, while some studies indicate a higher risk of non-contact ACL injuries during the mid-luteal phase, overall evidence remains inconclusive. Knee laxity varies across the menstrual cycle, affecting knee joint loading, but the quality of supporting evidence is limited. Practitioners should approach physical preparation, injury prevention, and screening with caution. Monitoring knee laxity could serve as a valuable tool in assessing ACL injury risk [56]. Pathophysiological changes in tissues surrounding the ACL, including cartilage and bone, can also impact its anatomical position and biomechanical function. For example, circumferential swelling or the formation of bone spurs (osteophytes) can lead to ACL impingement and damage. Dhillon *et al.* reported a case where hypertrophy of the resident’s ridge compressed and compromised the ACL [57]. Similarly, Cha *et al.* found a link between ACL adhesive disease and a narrow, concave intercondylar notch morphology, suggesting that a narrower notch increases the risk of ACL damage due to potential collisions during activity [58]. Additionally, conditions such as ectopic bone ossification and synovial cysts on the ACL can obstruct its movement [59, 60]. Detecting and assessing these conditions before surgery is crucial for developing appropriate surgical strategies.

The anatomy of the knee joint can influence the occurrence of postoperative complications. Adhesion syndrome, a complication that may arise after ACL reconstruction, is believed to be associated with smaller or abnormally shaped intercondylar notches where the ACL is placed during surgery [61]. Studies by Kim *et al.* examined how implant placement during surgery can affect adhesion formation. Therefore, selecting bone tunnel locations that follow the original ACL path and avoiding areas prone to impingement is critical in preventing adhesion syndrome [62].

Degenerative changes in the cartilage surrounding the knee joint may accelerate after an ACL injury, increasing the risk of osteoarthritis (OA). Post-traumatic osteoarthritis following ACL reconstruction is a common concern, with studies indicating that altered joint mechanics and increased wear on the articular cartilage contribute to cartilage degradation over time. Factors such as improper alignment during surgery, altered load distribution, and residual instability may further exacerbate cartilage damage and impact long-term functional outcomes [63, 64]. Synovial inflammation following ACL injury or surgery can trigger an excessive healing response, leading to scar tissue formation that hinders ACL recovery. In some cases, excessive scarring can cause joint stiffness and restricted range of motion, a condition known as arthrofibrosis. Pro-inflammatory cytokines, including IL-1β and TNF-α, may further exacerbate synovial inflammation, complicating recovery. Managing inflammation through pharmacological treatments or physical therapy may help reduce the risk of excessive scarring and improve surgical outcomes [65, 66].

### 3.2 Influence of Age and Developmental Stage (Children, Adolescents and the Elderly)

Beyond gender, age and developmental stage significantly influence the relationship between the ACL and surrounding skeletal structures. As previously mentioned, ongoing skeletal development in children and adolescents can cause the ACL's tibial insertion site to deviate from its normal anatomical position [28, 34]. Additionally, the intercondylar notch width index during this stage is notably smaller compared to adults [36]. These anatomical variations contribute to the increased risk of ACL injury in younger individuals and necessitate specific considerations for tunnel placement during reconstructive surgery. During adolescence, changes occur in ligament elasticity, tensile strength, and the ACL's total stress tolerance. This may contribute to the increased risk of injury in younger groups participating in physical activities. Incorporating neuromuscular and balance training programs throughout development phases may reduce these risks by improving joint stability, coordination, and strength, thereby preventing ACL injuries before skeletal maturity [67, 68]. As people age, the biomechanical qualities of ligaments, especially the ACL, degrade, resulting in decreased elasticity, lower collagen density, and overall ligament structural weakness. Reduced muscle strength, longer neuromuscular reaction times, and impaired balance all contribute to an increased risk of ACL damage or surgical intervention [69, 70]. Understanding these biomechanical alterations may help improve injury prevention methods and surgical outcomes for older patients undergoing ACL reconstruction.

Furthermore, studies have shown that the intercondylar notch volume also diminishes in the elderly population [71]. This decrease might be linked to the higher prevalence of degenerative osteoarthritis. Research suggests that intercondylar notch narrowing is a common occurrence among patients with degenerative knee conditions [72]. When ACL reconstruction becomes necessary in such cases, a narrowed notch may restrict femoral tunnel placement, necessitating a surgical plan customized to the patient's unique anatomical characteristics.

Neuromuscular control and coordination vary significantly with age, influencing ACL injury risk. In younger individuals, particularly adolescents, neuromuscular development is still incomplete, and motor control during high-intensity activities such as jumping or pivoting is not fully refined. This often results in poor landing techniques, increasing stress on the ACL. Implementing age-appropriate neuromuscular and balance training programs aimed at improving motor coordination and dynamic balance could help reduce injury risks in this population. Conversely, aging leads to a decline in neuromuscular efficiency, characterized by reduced proprioception and slower reaction times, both critical for joint stability. Elderly individuals may experience heightened knee instability, increasing their susceptibility to injury. Customized physical therapy and neuromuscular re-education programs can enhance recovery outcomes for older patients following ACL injuries [73, 74].

Bone mineral density plays a crucial role in ACL injury and recovery. In pediatric and adolescent patients, growing bones with open growth plates are more prone to injury, often resulting in avulsion fractures [75]. Transphyseal ACL reconstruction in children carries a 24% risk of minor leg length discrepancies. While the surgery can cause some angulation changes in the femur and tibia, these tend to balance each other out. Growth disturbances are not linked to a specific age within the pediatric group. Despite this risk, the procedure generally leads to good clinical outcomes, including improved function, stability, and patient satisfaction [76].

ACL reconstruction in children presents unique challenges due to ongoing bone development, making it a critical area of medical research. Price *et al.* conducted a multi-center study, collecting data from hospitals with varying medical capabilities. They rigorously screened cases and categorized children by infancy, preschool, and school age. By evaluating age-related surgical tolerance, monitoring key intraoperative and postoperative indicators, and comparing single-bundle and double-bundle reconstruction techniques, their research provided valuable insights into optimizing ACL reconstruction strategies for young children, offering a strong foundation for clinical practice [77]. Migliorini *et al*. conducted a comprehensive review of authoritative databases to compare the outcomes of ACL reconstruction using allografts and autografts. Their analysis revealed that while allografts offer easier surgical handling, they are associated with a higher risk of rejection. In contrast, autografts eliminate rejection concerns but come at the cost of additional donor site trauma [78]. Gausden *et al.* introduced 3D MRI dynamic imaging technology and combined it with biomechanical principles to optimize the surgical plan, improve knee joint stability, and reduce the impact on bone growth [79]. Yang *et al.* constructed a multi-dimensional Child Opportunity Index evaluation system and found that social-environmental factors affected postoperative rehabilitation [80]. Mallinos *et al.* reviewed a large number of clinical cases of adolescent patients, summarized common injury risk points in growth plate surgery, such as improper instrument operation and inaccurate fixation positions, and proposed the use of biodegradable materials for fixation to avoid secondary surgery. At the same time, it emphasized the formulation of personalized rehabilitation plans based on the growth and development stages, surgical methods, and physical recovery abilities of adolescents to assist in rehabilitation [81].

These studies, from multiple aspects such as surgical methods, graft selection, and social factors, provide references for the clinical practice and subsequent research of anterior cruciate ligament reconstruction surgery in children, helping to improve the treatment level and the quality of life of children. This necessitates careful consideration of treatment strategies based on skeletal maturity and bone strength. In contrast, elderly individuals with osteoporosis or osteopenia may face challenges with graft fixation and healing after ACL reconstruction due to poor bone quality [82]. Therefore, assessing bone health and implementing bone-strengthening interventions can enhance post-operative outcomes in both pediatric and elderly populations [83].

Age-specific surgical techniques for ACL restoration differ owing to the distinct problems that each age group faces. Adolescent patients face unique challenges in ACL reconstruction due to the presence of open growth plates in the proximal tibia and distal femur. Delaying the reconstruction until skeletal maturity can protect the epiphysis, but it increases the risk of secondary injuries. On the contrary, early surgical intervention can restore knee joint stability, yet it poses potential risks for growth disorders. Both conservative and surgical treatments have their own advantages and disadvantages. Finite element modeling (FEM) can optimize surgical techniques, reduce iatrogenic injuries, and improve the prognosis. Although it has great potential, its clinical application is insufficient. This article emphasizes that FEM should be incorporated into pediatric ACL care, providing a decision-making framework for clinicians and pointing out future research directions in pediatric ACL reconstruction [81].

Advances in surgical procedures and imaging have improved the accuracy of ACL operations in this population, lowering the likelihood of long-term problems [84, 85]. In contrast, older individuals often have additional comorbidities, such as osteoarthritis, which may accompany ACL injuries. These patients may need a more extensive treatment strategy that includes both ACL repair and management of joint degeneration. In other circumstances, partial or complete knee replacement may be a better option since surgeons must carefully weigh the advantages of ACL restoration against possible drawbacks, such as delayed recovery and greater complication rates in this group [86, 87].

Therefore, anatomical variations across different ages and developmental stages can significantly impact ACL injury risk assessments and influence the selection of surgical strategies for reconstruction. Establishing standardized reference values and developing individualized diagnostic and treatment plans tailored to specific patient populations are crucial considerations. Rehabilitation protocols must be tailored to different age groups. Younger patients may benefit from more aggressive, sport-specific rehabilitation programs to return them to athletic activities safely, while older patients may require more conservative rehabilitation to account for reduced healing capacity, comorbidities, and other age-related factors. Monitoring bone health, muscle atrophy, and overall physical activity levels is essential in designing a successful rehabilitation plan [88].

### 3.3 Risk Associated with the Bone Morphology of the Posterior Tibial Slope and Meniscal Injuries

While the intercondylar notch and ACL insertion site are key areas of interest, other skeletal structures within the knee joint also play a role in ACL pathology, as illustrated in Fig. (**5**). For example, the posterior tibial slope angle of the tibia exhibits a crucial relationship with the ACL in terms of both anatomical position and biomechanical function. An increased lateral posterior tibial slope (LPTS) is significantly associated with a higher risk of lateral meniscal injury in patients with acute non-contact ACL rupture. The study reveals that elevated LPTS induces abnormal anterior inclination of the tibial plateau, which intensifies shear stress on the lateral meniscus (particularly the posterior horn) during ACL rupture, thereby increasing its tear incidence. The authors emphasize that such anatomical variations may predispose to combined meniscus-ligament injuries through biomechanical mechanisms (such as uncontrolled anterior tibial translation). They recommend preoperative imaging evaluation of LPTS to guide personalized surgical decision-making (combined meniscal repair or corrective osteotomy) and propose dynamic stability training protocols for high-risk patients to optimize outcomes. The findings suggest that correlations between anatomical parameters and soft tissue injuries should be incorporated into comprehensive ACL injury assessment frameworks [89].

The findings underscore the critical importance of preserving meniscal integrity during and after ACL reconstruction to prevent subsequent knee instability and degenerative changes. Since this anatomical feature varies among individuals, it must be fully considered during pre-surgical planning and post-injury risk assessments. In addition, pathological changes such as synovial hypertrophy and bone cysts can also alter the anatomical position of the ACL graft, potentially leading to postoperative complications [48, 50]. These research results indicate that the factors influencing the pathology of the ACL are multifaceted and extend far beyond the simple intercondylar notch and ACL insertion site. When conducting clinical assessments and formulating surgical strategies, it is of great significance to comprehensively consider the potential impacts of other skeletal structures within the knee joint for accurately diagnosing the condition and formulating reasonable treatment plans.

## 4 PREOPERATIVE ASSESSMENT AND SURGICAL STRATEGIES

### 4.1 Imaging Assessment (MRI, CT, 3D Reconstruction)

The intricate relationship between ACL pathology and skeletal anatomy underscores the importance of both preoperative imaging assessments and the intraoperative use of anatomical landmarks for successful surgical outcomes.

Traditional X-rays, CT scans, and MRIs are the primary imaging tools for ACL evaluation. MRI excels at visualizing soft tissues like cartilage and ligaments, allowing for detailed assessment of ACL damage and morphological changes [90, 91]. Conversely, CT scans provide high-resolution images of bone structures, facilitating the measurement of critical parameters like intercondylar notch volume. Technological advancements have popularized 3D imaging reconstruction techniques, enabling dynamic analysis of the relationship between the ACL and surrounding skeletal structures from various angles [47, 48].

Furthermore, innovative diagnostic frameworks are emerging. For example, Dias *et al.* proposed using the Inner Distance Shape Context (IDSC) to analyze femoral notch curvature in MRI images for ACL injury risk assessment. This method can differentiate between healthy and injured knees with an accuracy of around 66%, and further optimization can potentially minimize false positives and negatives [17]. Additionally, Li *et al.* developed a deep-learning MRI segmentation model that automatically measures intercondylar notch volume and explores its association with ACL injury. Analyzing MRI data from both injured and healthy patients, this model demonstrated exceptional performance [20]. These approaches offer valuable tools for quantitative assessment of anatomical parameters, paving the way for more effective integration of artificial intelligence (AI)-powered imaging technologies into clinical diagnosis and preoperative planning.

### 4.2 Intraoperative Anatomical Landmarks and Tunnel Positioning Optimization

During ACL reconstruction, anatomical landmarks serve as essential guides for precise femoral and tibial tunnel placement. Yonetani *et al.* introduced the “support slope” theory, highlighting the anterolateral inclination of the tibial intercondylar eminence as a key reference for tibial tunnel drilling [25]. Similarly, Li *et al.* proposed using the femoral trochlea edge to guide femoral tunnel placement, minimizing the risk of impingement against the intercondylar notch sidewall [45]. Further systematic analyses of anatomical landmarks at ACL insertion sites are expected to provide stronger evidence for optimizing tunnel positioning [92].

Advancements in imaging technologies continue to refine our understanding of the relationship between ACL pathology and skeletal anatomy. Intraoperative anatomical landmarks, combined with these modern tools, enhance surgical precision. Future developments will focus on integrating these approaches to enable more individualized and highly accurate ACL reconstruction procedures.

## 5 LIMITATIONS, CHALLENGES AND FUTURE PROSPECTS

Despite significant advancements in ACL reconstruction and rehabilitation, several limitations affect the interpretation and generalizability of current findings. Variability in skeletal anatomy across different populations makes it challenging to develop universally applicable surgical and rehabilitation protocols. Additionally, inconsistencies in imaging techniques and measurement methodologies introduce variability in ACL morphology assessments, highlighting the need for standardized approaches. Short- and medium-term follow-up periods in many studies also limit our understanding of long-term surgical outcomes and the impact of anatomical variations on knee stability.

While traditional rehabilitation methods have shown progress, Piedade *et al.* mentioned that personalized programs still require further refinement to better align with functional recovery goals [93]. This need for optimization is particularly critical in complex cases, such as pediatric ACL injuries. Dunn *et al.* highlighted the delicate balance between early surgical intervention and delayed or non-surgical treatment in young patients, where the risk of future knee instability must be weighed against potential damage to growth plate development [94].

Similarly, managing severe injuries, such as combined anterior/posterior cruciate ligament tears with posterolateral corner (PLC) damage, remains a challenge. Rochecongar *et al.* underscored the uncertainties in surgical and rehabilitation outcomes for these injuries [95]. Additionally, predicting recovery outcomes post-ACL reconstruction is complicated by the limitations of current assessment tools. Martin *et al.* identified a “ceiling effect” in available databases, making it difficult for clinicians to accurately forecast recovery potential and tailor post-surgical care effectively [96].

ACL injuries result from a complex interplay of anatomical, biomechanical, and environmental factors. However, much of the existing research focuses on isolated parameters, making it challenging to fully understand their combined effects. ACL injuries result from a complex interplay of anatomical, biomechanical, and environmental factors. However, much of the existing research focuses on isolated parameters, making it challenging to fully understand their combined effects. Artificial intelligence (AI) is transforming ACL injury management by providing innovative solutions in diagnosis, surgical planning, and rehabilitation. AI’s ability to personalize treatment plans, particularly through the use of big data, has been recognized as a key factor in optimizing rehabilitation strategies and improving patient outcomes.

AI-driven models also enhance injury prevention through risk factor analysis and personalized interventions. A deep learning model effectively assists clinicians in diagnosing ACL ruptures with expert-level accuracy and efficiency [97]. By analyzing data from various sources, AI-powered prediction tools can identify individuals at higher risk of ACL injuries, allowing for early intervention strategies. This proactive approach helps reduce the incidence of ACL injuries, particularly among athletes, by optimizing training regimens and movement mechanics to minimize strain on the ligament. Despite its potential, the widespread clinical adoption of AI-driven models and advanced imaging techniques faces several obstacles, including accessibility, high costs, and the need for specialized expertise. However, by integrating predictive algorithms with patient-specific data, AI can significantly enhance precision in injury management, reduce the risk of re-injury, and contribute to better long-term recovery outcomes. As technology continues to evolve, overcoming these barriers will be essential to fully harness AI’s potential in ACL treatment, rehabilitation, and injury prevention [19, 98].

The ACL reconstruction technique has evolved over a century, progressing from early empirical attempts (such as iliotibial band grafts, metal wire substitutes) to precision medical practices grounded in anatomical and biomechanical principles [99]. Recent breakthroughs in multidimensional technological innovations have transformed the field: 3D-printed guides for personalized tunnel positioning have significantly enhanced intraoperative precision [100]. AI-integrated preoperative imaging systems optimize surgical planning; minimally invasive arthroscopy combined with anatomical double-bundle reconstruction and dynamic stabilization techniques better replicate native ACL biomechanics; bioabsorbable implants, autografts/allografts, and synthetic ligaments improve graft durability; biological augmentation strategies (such as platelet-rich plasma, stem cell therapy) and remnant-preserving techniques promote tendon-bone healing; and biomechanics-driven rehabilitation protocols accelerate functional recovery. These advances have elevated long-term ACL reconstruction success rates from approximately 60% in early stages to 85%–95% today [101]. Looking ahead, the integration of AI navigation, 3D-printed custom instruments, and tissue engineering promises to further advance ACL reconstruction toward intelligent, precision-oriented, and biologically integrated approaches, ensuring enhanced functional restoration and long-term prognosis for patients [102].

## CONCLUSION

This review comprehensively examines the intricate relationship between ACL pathology and the surrounding skeletal structures of the knee joint, particularly the femur and tibia, providing valuable insights for clinical diagnosis and treatment. The key content can be summarized as follows:

- **Femoral Intercondylar Notch Morphology:** The size and shape of the femoral intercondylar notch significantly influence ACL injury risk. Narrowing, concavity, or reduced notch volume increases the likelihood of ACL impingement and injury. Advanced three-dimensional imaging and intelligent segmentation algorithms are expected to improve the accuracy of notch parameter measurements, enabling individualized ACL injury risk assessments.
- **Tibial Insertion Site Assessment:** Accurate evaluation of the ACL's tibial insertion site is critical for determining the angle and approach of the tibial bone tunnel. Local injuries, such as tibial tuberosity fractures, can impair ACL function and must be addressed preoperatively. Variations in bone epiphyseal development across age groups further necessitate tailored surgical adjustments.
- **Structural Parameters of the ACL:** Parameters such as length, cross-sectional area, and imaging signal intensity are closely linked to surrounding skeletal anatomy and serve as indicators of ACL structural and functional integrity. Predicting ACL structural parameters using skeletal measurements facilitates individualized biomechanical assessments.
- **Surgical Tunnel Placement**: Optimal positioning of femoral and tibial tunnels in ACL reconstruction is essential to replicate the ACL's anatomical path and avoid mechanical obstructions. Advanced three-dimensional imaging technologies provide precise data for quantitatively assessing implant positioning relative to skeletal structures.
- **Impact of other Skeletal Structures:** Pathological changes in structures such as the lateral condyle transverse ridge and posterior oblique line of the tibia may affect ACL positioning and biomechanics, requiring consideration in preoperative planning.
- **Individualized Assessment:** Variations in skeletal anatomy across genders and age groups necessitate tailored surgical planning. Children and adolescents require consideration of bone epiphyseal development, while elderly patients may face challenges such as intercondylar notch narrowing.
- **Technological Advancements:** Preoperative imaging assessments and intraoperative anatomical landmarks guide ACL insertion site localization and tunnel drilling. High-resolution three-dimensional imaging, artificial intelligence segmentation, and refined anatomical studies enhance precision in diagnosis and surgical planning.

The relationship between ACL pathology and surrounding skeletal structures highlights the importance of a thorough understanding of knee anatomy in clinical practice. This knowledge is critical for assessing ACL injury risk, optimizing surgical pathways, and improving patient outcomes. By integrating advanced imaging technologies and anatomical studies, clinicians can develop more precise and individualized diagnostic and treatment plans, ultimately enhancing surgical efficacy and reducing the risk of re-injury.

While significant progress has been made in understanding the relationship between ACL pathology and skeletal anatomy, several areas require further exploration. Future research should focus on the development of standardized protocols for three-dimensional imaging and AI-based segmentation to improve the accuracy and reproducibility of anatomical measurements. Additionally, long-term studies are needed to evaluate the impact of individualized surgical planning on patient outcomes, particularly in diverse populations such as pediatric and elderly patients. Furthermore, the influence of dynamic factors, such as joint loading and muscle activity, on ACL biomechanics remains underexplored. Addressing these gaps through multidisciplinary collaboration and technological innovation will be essential for advancing ACL injury prevention, diagnosis, and treatment.

## Figures and Tables

**Fig. (1):**
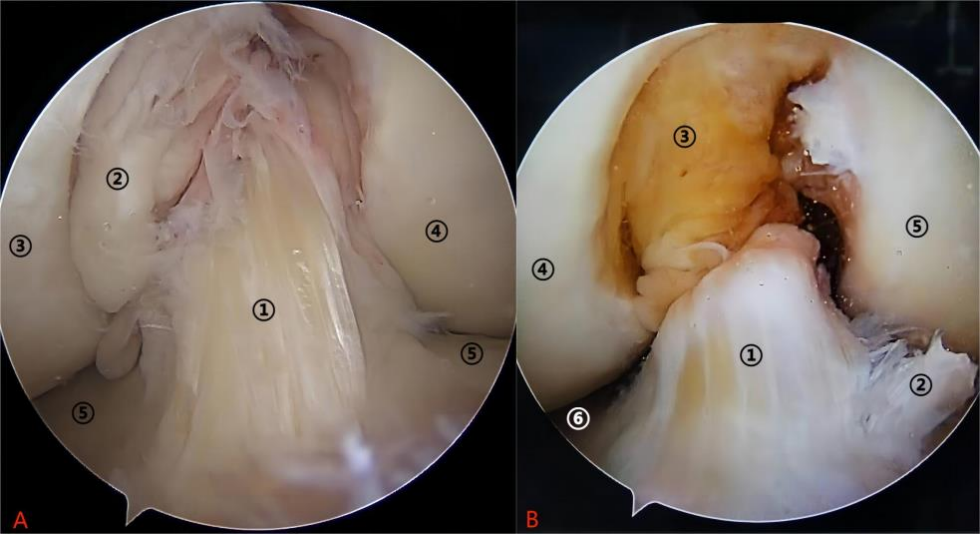
Arthroscopic view of a normal anterior cruciate ligament and a ruptured anterior cruciate ligament. **Left panel A shows:** (1) Normal anterior cruciate ligament (ACL); (2) Posterior cruciate ligament (PCL); (3) Medial femoral condyle; (4) Lateral femoral condyle; (5) Tibial plateau. **Right panel B** shows: (1) Ruptured anterior cruciate ligament; (2) Fibrous tissue of the ruptured ACL; (3) Posterior cruciate ligament; (4) Medial femoral condyle; (5) Lateral femoral condyle; (6) Tibial plateau. (Source: Radiology Department, Baise People's Hospital, Guangxi, China).

**Fig. (2):**
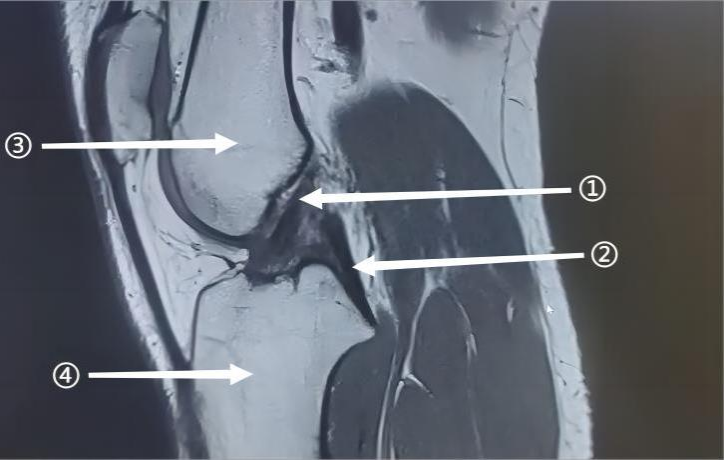
MRI sagittal view of the normal anterior cruciate ligament pathway. The figure shows: (1) Anterior cruciate ligament; (2) Posterior cruciate ligament; (3) Femur; (4) Tibia. (Source: Radiology Department, Baise People's Hospital, Guangxi, China).

**Fig. (3):**
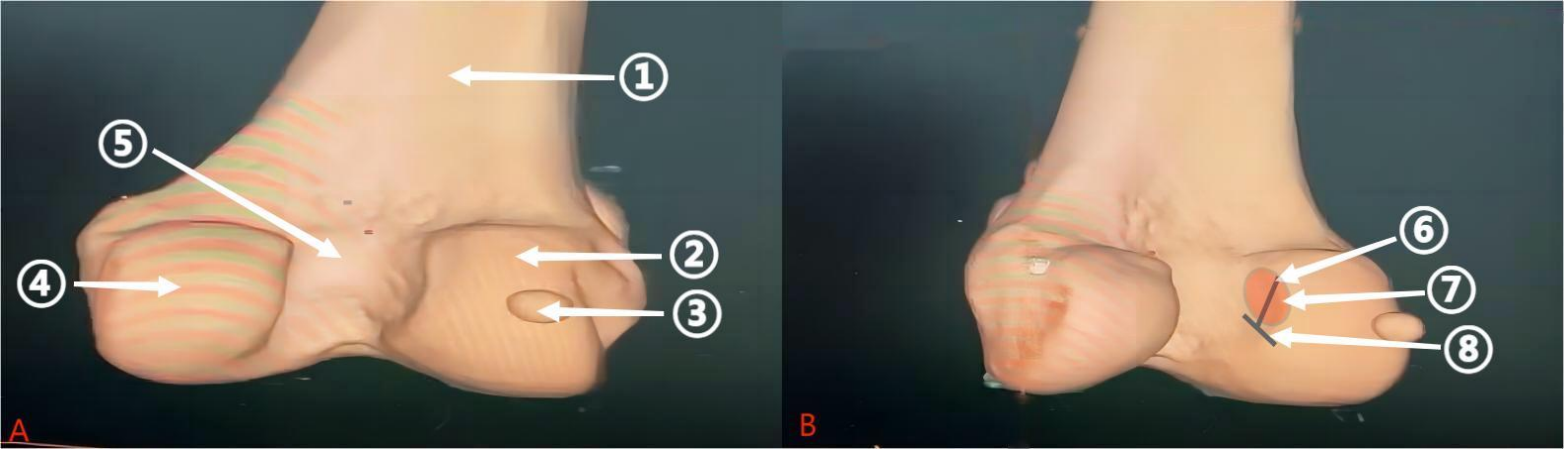
3D CT of the anterior cruciate ligament at the femoral footprint area. Left panel A shows: (1) Femur; (2) Lateral femoral condyle; (3) Patella; (4) Medial femoral condyle; (5) Intercondylar notch; (6) Bifurcate ridge; (7) Red area represents the schematic of the ACL origin footprint on the femur; (8) Lateral intercondylar ridge. (Source: Radiology Department, Baise People's Hospital, Guangxi, China).

**Fig. (4):**
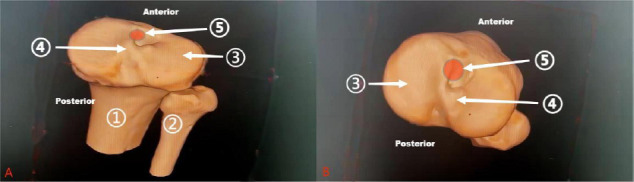
3D CT of the anterior cruciate ligament at the tibial plateau footprint area. Left panel A shows: (1) Tibia; (2) Fibula; (3) Tibial plateau; (4) Intercondylar eminence of the tibia; (5) Red area represents the schematic of the ACL insertion footprint on the tibial plateau. (Source: Radiology Department, Baise People's Hospital, Guangxi, China).

**Fig. (5):**
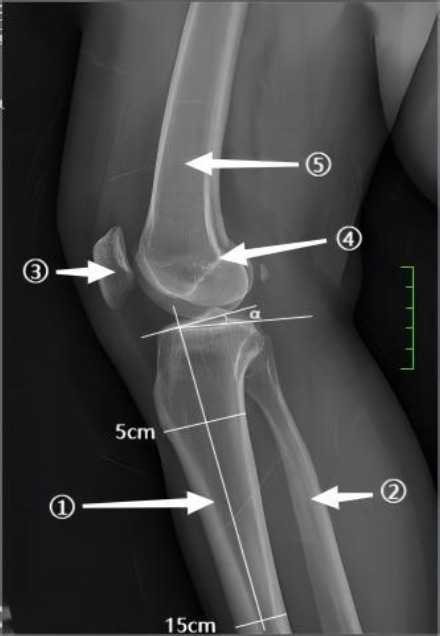
X-ray lateral view of the knee joint. The figure shows: (1) Tibia; (2) Fibula; (3) Patella; (4) Blumensaat's line; (5) Femur; ∠α (the posterior tibial slope angle) =10.7°. (Source: Radiology Department, Baise People's Hospital, Guangxi, China).

## DECLARATION

The english language of the article was improved with chatgpt. The author drafted, reviewed, and revised the manuscript and retains sole responsibility for the content presented in this publication.

## LIST OF ABBREVIATIONS

ACL: Anterior Cruciate Ligament
OA: Osteoarthritis
FEM: Finite Element Modeling
LPTS: Lateral Posterior Tibial Slope
PLC: Posterolateral Corner
AI: Artificial Intelligence
